# IgE alone promotes human lung mast cell survival through the autocrine production of IL-6

**DOI:** 10.1186/1471-2172-9-2

**Published:** 2008-01-23

**Authors:** Glenn Cruse, Sarah Cockerill, Peter Bradding

**Affiliations:** 1 Institute for Lung Health, Department of Infection, Immunity and Inflammation, University of Leicester and Warwick Medical School, University of Leicester, University Road, Leicester, LE1 9HN, UK; 2 Department of Respiratory Medicine, University Hospitals of Leicester, Glenfield General Hospital, Groby Road, Leicester, LE3 9QP, UK; 3 Department of Cell Physiology and Pharmacology, University of Leicester, University Road, Leicester, LE1 9HN, UK

## Abstract

**Background:**

Mast cells play a key role in asthma and recent evidence indicates that their ongoing activation in this disease is mediated, in part, *via *IgE in the absence of antigen. In this study we have examined whether IgE alone enhances human lung mast cell (HLMC) survival.

**Methods:**

Purified HLMC were cultured for 4 weeks and survival assays then performed over 10 days following cytokine withdrawal in the presence or absence of human myeloma IgE. Quantitative real time RT-PCR was carried out to examine IL-6 mRNA expression and IL-6 protein was measured in HLMC supernatants by ELISA.

**Results:**

IgE alone promoted the survival of HLMC in a dose-dependent manner following cytokine withdrawal. IgE-induced survival was eliminated with the addition of neutralising anti-IL-6 antibody but not by the addition of neutralising anti-stem cell factor. IgE sensitisation initiated profound upregulation of IL-6 mRNA in HLMC, and IL-6 concentrations were also raised in the culture supernatants of IgE-exposed cells.

**Conclusion:**

These data taken together suggest that IgE in the absence of antigen promotes HLMC survival through the autocrine production of IL-6. This provides a further mechanism through which IL-6 and IgE contribute to the pathogenesis of asthma, and through which anti-IgE therapy might achieve its therapeutic effect.

## Background

Mast cells play a key role in many physiological and pathophysiological processes. They contribute to the maintenance of tissue homeostasis, wound repair [1,2] and revascularisation [3], as well as exerting protective roles in both acquired and innate immune responses to bacterial infection [4]. However, mast cells are synonymous with allergy due to the destructive effects of their mediators when released in excess through IgE-dependent mechanisms. In asthma, mast cells infiltrate the airway smooth muscle (ASM) bundles, airway epithelium and submucosal glands, placing them in direct contact with these dysfunctional airway elements [5].

Mast cells can be activated by many diverse stimuli leading to mediator release but allergen-dependent activation occurs predominantly through the high affinity IgE receptor complex (FcεRI) following aggregation of allergen-specific IgE bound to FcεRI (Reviewed in [6,7]). IgE binding to FcεRI in the absence of antigen has long been considered to represent a passive sensitisation of mast cells. However, this view has been challenged due to increasing evidence that monomeric IgE binding to FcεRI initiates intracellular signalling events leading to distinct cellular responses [8-19]. IgE alone directly activates human lung mast cells (HLMC) leading to Ca^2+ ^influx and the release of histamine, leukotriene C_4 _(LTC_4_) and CXCL8 [8]. Thus increased IgE production in atopic asthma could directly contribute to the mast cell hypersecretion and prolonged activation evident within asthmatic bronchi [5].

Understanding the mechanisms of mast cell hyperplasia in diseased tissue structures is of interest because inhibiting this might offer new approaches to treatment. Increased mast cell recruitment by the asthmatic ASM for example appears to be one factor [20]. However enhanced mast cell survival might be a further factor. In rodents, IgE not only activates mast cells leading to mediator release, but also prolongs their survival through the autocrine production of survival-enhancing cytokines, particularly IL-3 [21]. IgE-dependent mast cell survival may therefore also be a factor contributing to the increased numbers of mast cells evident in key airway structures of the asthmatic airway.

In this study, we have tested the hypothesis that IgE alone enhances HLMC survival through the production of the survival enhancing cytokines IL-6 and stem cell factor. We demonstrate for the first time that monomeric IgE in the absence of antigen enhances HLMC survival, and that this effect is mediated, at least in part, through the autocrine production of IL-6.

## Results

### IgE alone promotes HLMC survival following cytokine withdrawal

**Figure 1:**
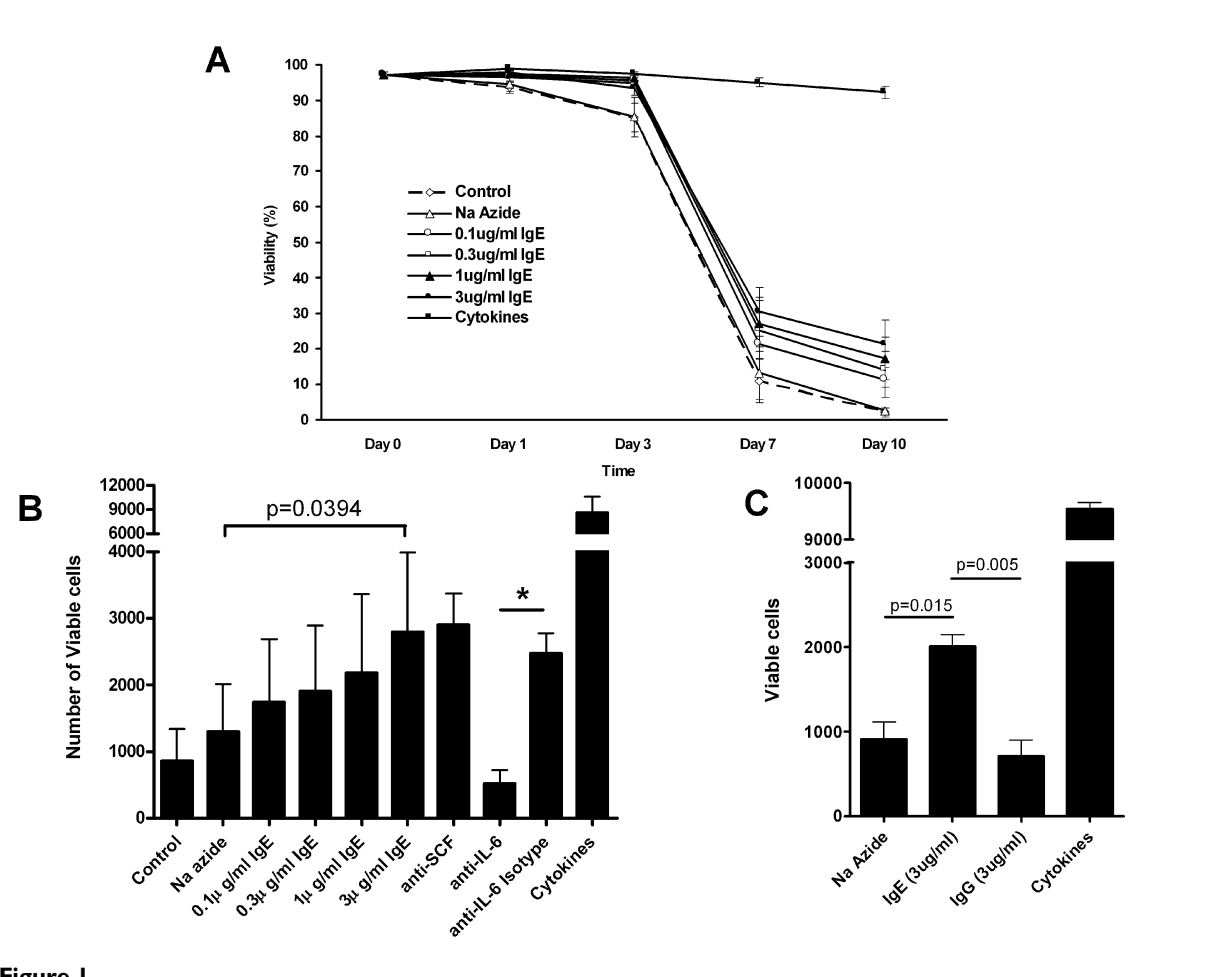
**IgE promotes the survival of cultured human lung mast cells through IL-6**. **A) **Survival assay time course following cytokine withdrawal. Human lung mast cells (HLMC) die rapidly following cytokine withdrawal and this cell death is attenuated with the addition of IgE. **B) **IgE-induced HLMC survival is eliminated with the addition of an anti-IL-6 antibody and unaffected with the addition of an anti-SCF antibody following 7 days of culture. **C) **IgG does not promote HLMC survival when compared to the sodium azide control. Data is presented as the mean ± SEM from 6 different donors performed in triplicate. Blocking experiments are the mean ± SEM from 3 donors. Data for the IgG control experiments are the mean ± SEM from 4 donors. Donors in **C **are different to those in **A **and **B**.

Human lung mast cells undergo apoptosis with SCF and IL-6 withdrawal [22]. We therefore tested the effects of IgE alone on mast cell survival following SCF, IL-6 and IL-10 withdrawal. Following cytokine withdrawal, there was evidence of a decrease in cell viability in the control cells, which contained no IgE, even as early as 24 hours which was significant by day 3 (Figure 1A) (p = 0.020, n = 6). There was a significant dose-dependent increase in HLMC viability with the addition of IgE by day 7 when compared to the sodium azide control (Figure 1A). Thus at day 7, HLMC % viability was 11.0 ± 6.0% in the control compared to 13.3 ± 7.5% with 0.00015% sodium azide (p = 0.3419, n = 6). With the addition of 0.1, 0.3, 1 and 3 μg/ml IgE, HLMC % viability was 21.3 ± 8.8, 25.4 ± 8.2, 26.9 ± 7.6 and 30.5 ± 7.0% respectively (Figure 1A) (p = 0.0397, p = 0.0056, p = 0.0214 and p = 0.0014 respectively, n = 6). Interestingly, we found there to be no significant difference between the sodium azide control and the cells alone at either days 1, 3, 7 or 10 (p = 0.3781, p = 0.9595, p = 0.3419 and p = 0.7462 respectively, n = 6).

In order to determine the mechanism of the enhancement of mast cell survival with IgE we next investigated the autocrine production of the pro-survival cytokines SCF and IL-6. There was absolutely no effect on survival with the addition of neutralising anti-SCF (1 μg/ml) or isotype control antibodies (Figure 1B). However, with the addition of neutralising anti-IL-6 (1 μg/ml) there was a marked decrease in the number of viable cells compared to the isotype control (Figure 1B). Thus in the isotype control, there were 2467 ± 309 viable cells at day 7 compared to 517 ± 204 viable cells with the addition of anti-IL-6 (Figure 1B) (p = 0.041, n = 3). As an additional control, we also investigated the effects of IgG on HLMC survival. Thus with the addition of 3 μg/ml IgE, there were 2008 ± 143 viable cells at day 7 compared to 712 ± 189 viable cells with the addition of 3 μg/ml IgG (Figure 1C) (p = 0.005, n = 4). Therefore, the addition of IgG did not affect HLMC survival when compared to the sodium azide control (712 ± 189 viable cells with 3 μg/ml IgG, compared to 906 ± 208 viable cells in the sodium azide control, Figure 1C, p = 0.515, n = 4).

### Quantitative real time RT-PCR

**Figure 2:**
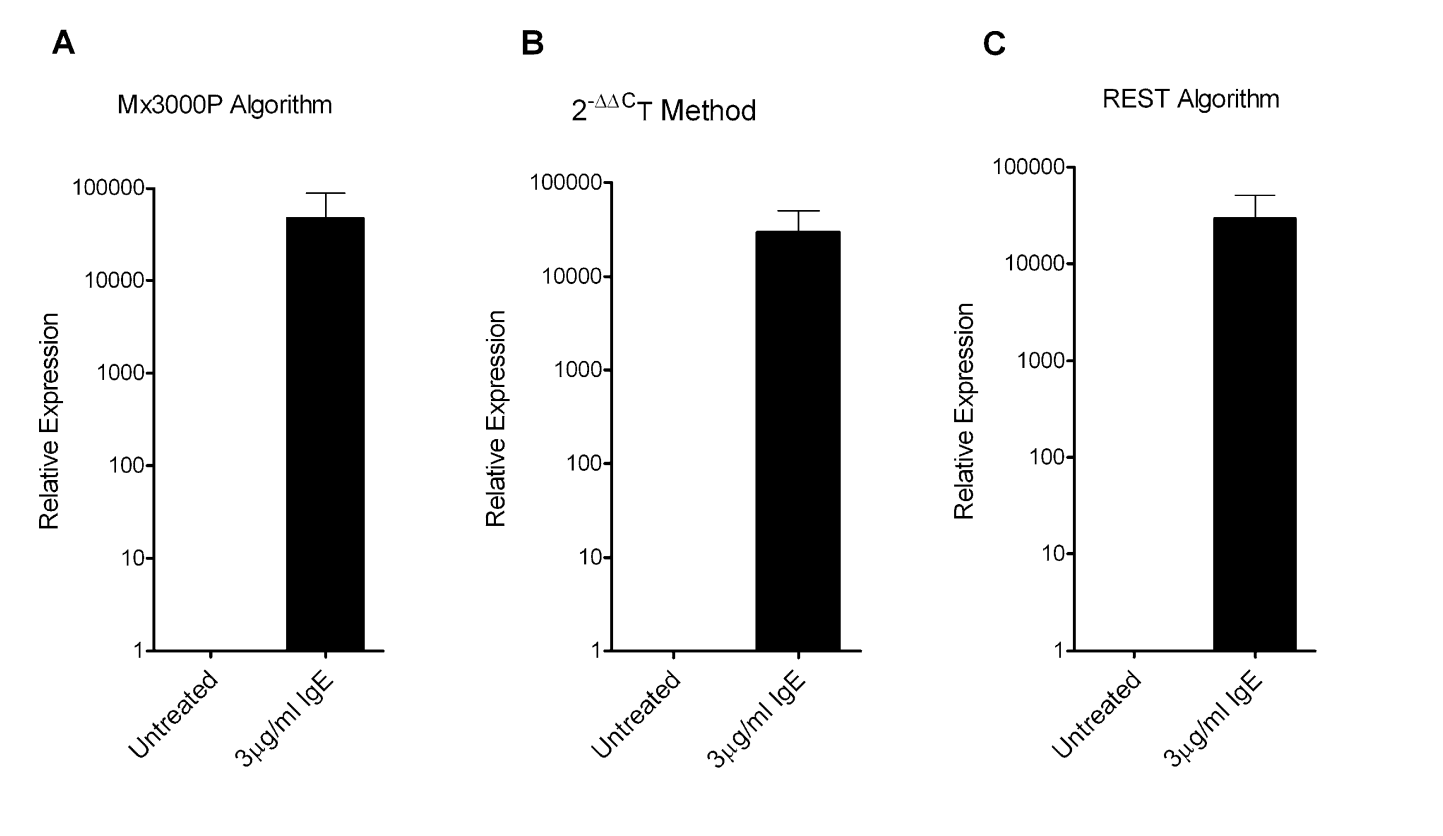
**Monomeric IgE induces the upregulation of IL-6 mRNA transcription in freshly isolated human lung mast cells**. Human lung mast cell IL-6 mRNA expression was determined using comparative quantitative real time RT-PCR and upregulation of expression was confirmed using three different algorithms for calculation of results.

**Figure 3:**
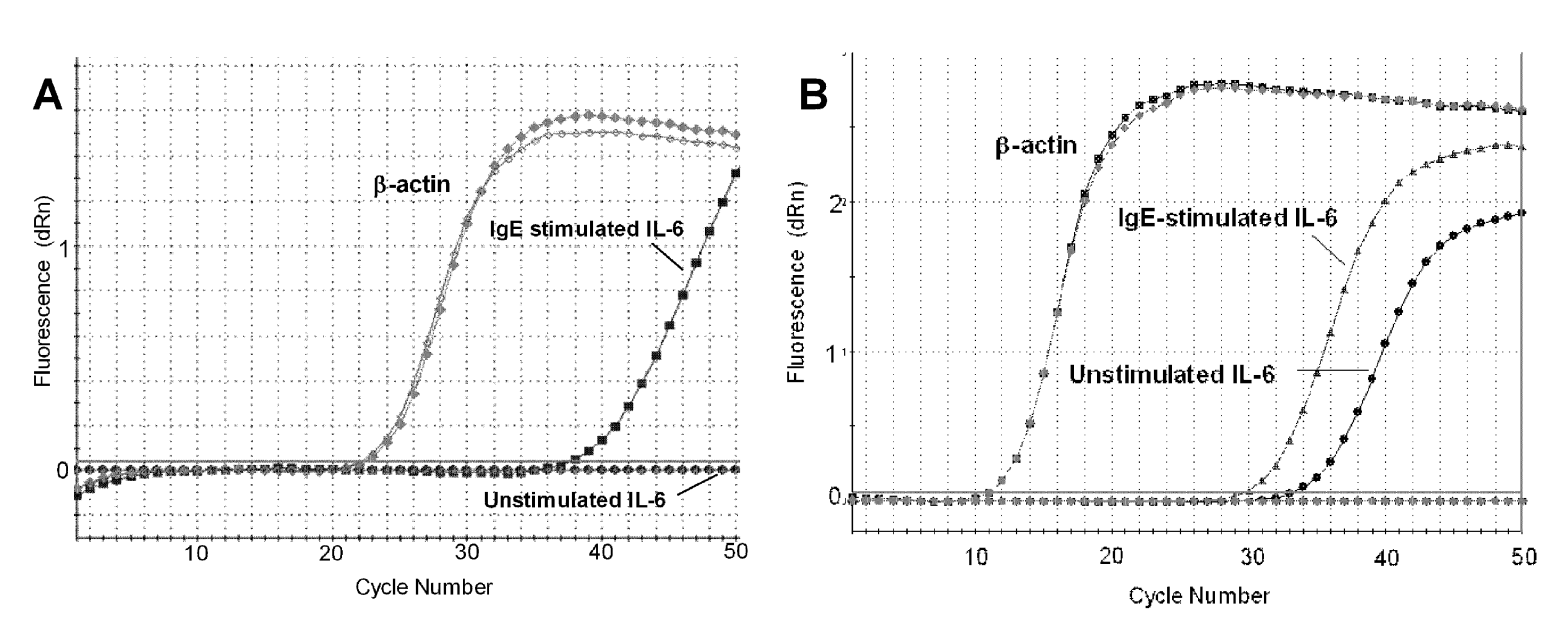
**Amplification plots for quantitative real time RT-PCR demonstrating the heterogeneity of IL-6 mRNA expression**. **A) **Amplification plot of a donor with low expression of IL-6 mRNA even after IgE stimulation and no C_t _in the control cells. **B) **Amplification plot of a donor with higher expression of IL-6 mRNA including constitutive expression in the unstimulated cells. All donors exhibited upregulation of mRNA expression with the addition of IgE.

In order to confirm that IL-6 expression in HLMC was upregulated with the addition of IgE alone, comparative quantitative real time RT-PCR was carried out and the relative expression of IL-6 mRNA in IgE (3 μg/ml)-stimulated cells compared to unstimulated cells. The relative expression of IL-6 mRNA in the IgE-stimulated cells was increased 46997 ± 41171 fold compared to control (Figure 2) (p = 0.043, n = 14) when calculated with the Mx3000P's built in algorithm with an adaptive baseline. The degree of upregulation was highly variable, but was consistently greater than the control which in 2 donors did not achieve C_t _even after 50 cycles (Figure 3). The 2 donors where the control reaction did not reach C_t _had a relative expression of 577000 and 80591, which accounts for the huge relative expression and variability when the mean is plotted. We therefore analysed the data using the median and range. The median (range) relative expression of IL-6 mRNA was 3.79 (1.01–577000) when compared to control.

Due to this inherent variability, and since the determination of relative expression can vary greatly depending on the algorithm used, we next confirmed the gene upregulation using the 2^-ΔΔCt ^method which has been validated as a good measure of relative gene expression [23]. The mean C_t Target _– C_t Calibrator _for IL-6 in the control was 17.93. With the addition of 3 μg/ml IgE, the mean C_t Target _– C_t Calibrator _was 13.62 (p = 0.018, n = 14). Thus using the 2^-ΔΔCt ^calculation, the relative expression of IL-6 following IgE stimulation was upregulated by 29445 ± 21013 (Figure 2), which is comparable to the data from the Mx3000P algorithm. The median (range) relative expression of IL-6 mRNA was 3.38 (1.0–267652) when compared to control using the 2^-ΔΔCt ^algorithm. For statistical analysis, C_t _data was imported into the Relative Expression Software Tool (REST 2005, version 1.9.12, Corbett Life Science) which uses a different algorithm. The relative expression using the REST algorithm was almost identical to the 2^-ΔΔCt ^algorithm with an upregulation of 29445 ± 21013 (Figure 2), which after bootstrapping with 50 000 iterations was significant (p = 0.043, n = 14).

**Figure 4:**
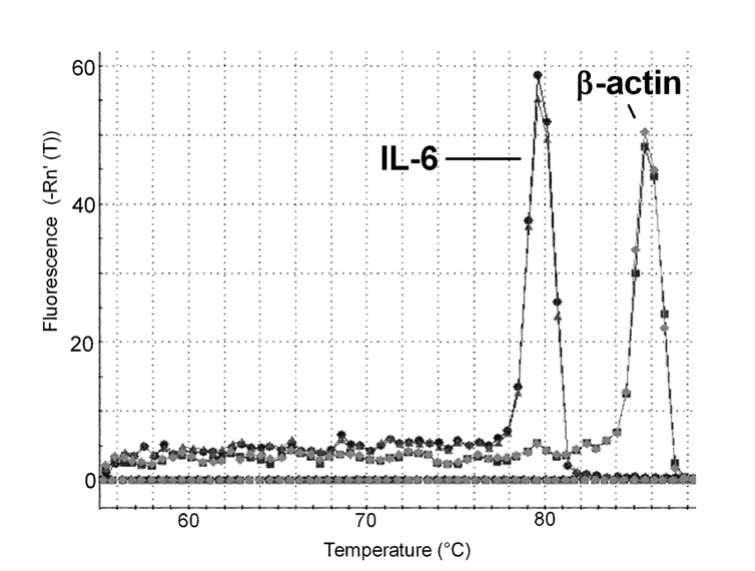
**SYBR Green melting curve for quantitative real time RT-PCR**. The melting curves for human lung mast cell QRT-PCR consistently gave a single peak with no evidence of non-specific amplification or primer-dimerisation. The graph is from a single experiment and representative of all donors.

**Figure 5:**
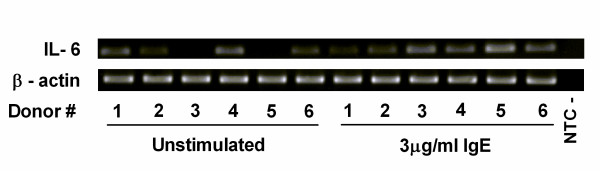
**PCR products from the quantitative PCR experiments run on an agarose gel**. PCR products from six donors were run on a 1.5% agarose gel to determine the size of the products. The product sizes corresponded to the expected size for both IL-6 and β-actin.

A dissociation curve was performed on all experiments and a distinct peak was observed consistently around 79.5°C for IL-6 and around 85.8°C for β-actin (Figure 4). QRT-PCR products were run on a 1.5% agarose gel to determine the product size and the IL-6 product was indeed 250 bp and β-actin was 310 bp (Figure 5). Bands were gel excised, purified and sequenced which confirmed specificity.

### Measurement of IL-6 release using ELISA

**Figure 6:**
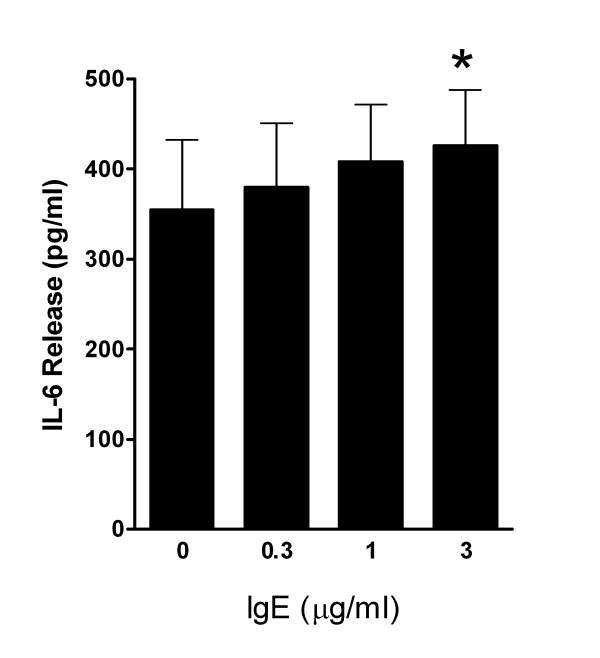
**Total IgE-induced IL-6 release into the supernatants at day 7 following cytokine withdrawal**. Data is presented as the mean ± SEM IL-6 release from six individual donors.

To confirm that the upregulation of IL-6 transcription with the addition of IgE was associated with an increased release of IL-6, we next measured the IL-6 released into the supernatants after 7 days of cytokine withdrawal. This revealed a dose-dependent increase in IL-6 release with the addition IgE. Thus with the addition of 3 μg/ml IgE, net IL-6 release was 71.3 ± 25.8 pg/ml (Figure 6) (p = 0.034, n = 6). Total IL-6 concentration with 3 μg/ml IgE was 426 ± 62 pg/ml of culture medium.

## Discussion

This study makes the novel observation that IgE in the absence of antigen promotes the survival of HLMC following cytokine withdrawal. This survival advantage is associated with increases in HLMC IL-6 mRNA expression and protein release, and is abrogated by neutralising antibody to IL-6 but not SCF. This suggests that IgE-dependent HLMC survival is mediated, in part, through the autocrine production of IL-6.

We have found that in the presence of clinically relevant concentrations of IgE (1 μg/ml = ~400 kU/L), HLMC survival is enhanced following growth factor withdrawal. Although SCF is the major growth and survival factor for HLMC, and is released by them in an autocrine manner [24], IgE-dependent survival was not accounted for via an SCF-dependent mechanism because neutralising SCF was without effect. In contrast, neutralisation of IL-6 completely inhibited the survival effect indicating that survival was mediated at least in part through IL-6. IL-6 is known to prevent apoptosis in HLMC and cord blood-derived mast cells (HCBMC) [25,26], and so this mechanism is entirely plausible. It is further supported by the fact that IL-6 mRNA expression was increased markedly in the presence of IgE and this was paralleled by a small but significant increase in the secretion of IL-6 protein into the HLMC culture supernatant. The concentration of IL-6 in the culture supernatant in the presence of 3 μg/ml IgE was approximately 426 pg/ml. This is not far below the optimal concentration of 1 ng/ml required to prevent apoptosis of HCBMC following growth factor withdrawal [25]. However, HLMC tend to aggregate in cell culture, and so are likely to be exposed to much higher local concentrations of HLMC-derived IL-6 than that present in the whole culture supernatant. However, there was also approximately 355 pg/ml of IL-6 in the control supernatant in which the HLMC had died, suggesting that although IL-6 promotes survival with IgE, a co-operative interaction with IgE-dependent signalling is also likely. Indeed, a study published while this paper was out for review demonstrated that monomeric IgE alone induced the upregulation of both mRNA and protein expression of the anti-apoptotic Bcl-xL protein, whilst down-regulating the expression of the pro-apoptotic proteins Puma and Bim [27].

A study of the effects of anti-IgE therapy (omalizumab) on airway inflammation in asthma identified a downward trend in both epithelial and lamina propria mast cell numbers of approximately 20% compared to control although this did not reach statistical significance [28]. However this study was underpowered with only 14 patients in the omalizumab group and 14 patients in the placebo group. There is therefore insufficient clinical data at present to confirm or refute the *in vivo *relevance of our *in vitro *findings regarding the ability of IgE to enhance mast cell survival. An important feature of mast cell activation in the presence of IgE alone is that while free IgE is present, intracellular signalling is sustained, whereas this ceases as soon as free IgE is removed [14]. The mechanisms behind this are still not known but indicate that this signalling mechanism is likely to be important *in vivo *where IgE exposure is continuous. Bearing in mind the strong correlation between serum IgE and the presence of asthma [29-32], it is conceivable that IgE contributes to the increased numbers of mast cells evident in the airway epithelium, mucosal glands and airway smooth muscle of asthmatic subjects [5].

We have demonstrated recently that IgE in the absence of antigen directly activates cultured HLMC leading to the release of histamine, and the production of LTC_4 _and CXCL8 [8]. The release of histamine and LTC_4 _was much greater in the presence of SCF, a vital growth and differentiation factor for human mast cells which also potentiates IgE-dependent mediator release [22,33]. However in the present study, when HLMC were incubated with IgE in the absence of any exogenous cytokines substantial IL-6 production was evident. This indicates that while SCF is required for substantial histamine release [8], monomeric IgE-induced cytokine production can proceed in it's absence. This is in keeping with 2 studies of human cord blood-derived mast cells (HCBMC) which failed to detect histamine release in the absence of SCF but did demonstrate the release of the chemokines CCL1, CCL2 and CXCL8 [15,34]. The study of HCBMC by Matsuda and colleagues also failed to find any survival effect with IgE [15]. One factor which might explain the difference in response between HLMC and HCBMC in terms of both histamine release and survival is the degree of surface FcεRI expression. FcεRI expression is relatively low on HCBMC [35], and demonstrates one example of the heterogeneity evident between mast cells from different tissues. Thus, mast cells with higher FcεRI expression appear to be more reactive to IgE, which may also contribute to the pro-survival effect evident in this study. A second factor of relevance may be the preparations of IgE used in the different studies. In mice, it has been demonstrated that some preparations of IgE are much more effective at promoting both degranulation and cytokine-dependent survival [10,16,36]. Although IL-6 is among those cytokines released by mBMMC, recent studies suggest that IL-3, a known growth factor for mouse BMMC, is the major cytokine involved in their protection from cell death [21].

Matsuda and colleagues found that the sodium azide present in their myeloma IgE preparation enhanced survival in the absence of IgE. For this reason, we ensured that all of our experimental conditions contained an equal amount of sodium azide, and included an appropriate sodium azide control. We also found there to be a small survival enhancing effect with the addition of sodium azide to the cells, but this was far less marked than that reported by Matsuda et al [15]. However the concentration of azide in their experiments was 2 fold higher than ours, although they showed there to be only around 6% difference in apoptotic cells between 0.0003% sodium azide and concentrations 100 fold less [15]. Therefore, small differences in sodium azide concentration are very unlikely to have any great effect on the survival data.

Mast cell signalling and responses to IgE alone raise the question as to whether the IgE preparations used are truly monomeric or just complexes of IgE which mimic receptor cross-linking with antigen, and whether the physiological concentrations of IgE used *in vitro *induce IgE to self aggregate at the receptor [37,38]. In fact, there is evidence that like receptor crosslinking with allergen, the signalling from IgE alone in the absence of allergen is also a result of receptor aggregation [10]. However, there are several lines of evidence which indicate that although receptor aggregation is initiated, distinct signalling pathways are recruited and that the effects of IgE alone are not an artefact. For example, although influx of extracellular Ca^2+ ^is a critical requirement for mediator release in mast cells activated by either IgE alone or by antigen, the channels carrying Ca^2+ ^into the cell following monomeric IgE stimulation appear to be different [13]. In addition, cytokine release induced by IgE alone is often much greater than that initiated in antigen-stimulated cells [11,17]. Furthermore, IgE induced Ca^2+ ^influx requires PKCβII whilst antigen-induced Ca^2+ ^influx does not [11]. Interestingly, HPLC purification of human myeloma monomeric IgE, which ensures that there are no multi/dimeric complexes, does not abrogate the biological response and, if anything, enhances it [15]. Furthermore, while IgE induces marked IL-6 production from mouse BMMC, IgE aggregates have no effect [17], confirming that mast cell responses to IgE are not due to IgE aggregates. There is therefore robust evidence that the *greater *the percentage of IgE monomers, the *greater *the biological response. All of these observations point towards distinct signalling pathways in mast cells activated by IgE alone versus antigen-dependent FcεRI cross-linking.

## Conclusion

In summary, we demonstrate that IgE in the absence of antigen promotes HLMC survival in a dose-dependent manner, and that this survival is markedly attenuated with the addition of neutralising anti-IL-6 antibody. Since IL-6 mRNA transcription is dramatically upregulated with the addition of IgE, and there is an increased release of IL-6 into the supernatant of IgE stimulated cells, we can conclude that the prosurvival effect of IgE in HLMC is due, at least in part, to the autocrine production of IL-6. This study provides a further mechanism through which IL-6 and IgE contribute to the pathogenesis of asthma, and through which anti-IgE therapy might achieve its therapeutic effect.

## Methods

### Purification and culture of human lung mast cells

All human subjects gave written informed consent and the study was approved by the Leicestershire Research Ethics Committee, UK. Lung tissue was obtained by surgical resection for bronchial carcinoma and mast cells isolated as described previously [39]. The final HLMC purity was >98% with cell viability >97% (monitored by exclusion of trypan blue).

Following isolation, HLMC were cultured in DMEM/Glutamax/HEPES containing 10% FBS, 1% MEM nonessential amino acids (all from Life Technologies), 1% antibiotic/antimycotic solution (Sigma-Aldrich), 100 ng/mL recombinant human (rh)SCF, 50 ng/mL rhIL-6 and 10 ng/mL rhIL-10 (R&D, Abingdon, UK) at 37°C in a humidified incubator flushed with 5% CO_2 _for a minimum of 4 weeks prior to experiments. Half of the medium was changed every 7 days. HLMC purity remained unchanged during the culture period.

### HLMC survival assay

Long term cultured HLMC were counted using an haemocytometer and cell viability assessed using exclusion of trypan blue stain. HLMC were washed to remove the cytokines present in the culture medium. 1 × 10^4 ^HLMC were plated into each well of a 96 well cell culture plate in 50 μl of DMEM/10% FBS. 50 μl of 2× the final concentration of human myeloma IgE (Calbiochem-Novabiochem, Nottingham, UK) or medium alone was added. IgE was centrifuged at 14000 g for 20 min to remove any large aggregates [8,15]. IgE preparations (100 μg/ml) contain 0.005% sodium azide. Therefore, dilutions of IgE were prepared to give a final concentration of sodium azide of 0.00015% in all conditions (including a control) except for the cytokine control (containing 100 ng/ml SCF, 50 ng/ml IL-6 and 10 ng/ml IL-10) and the no-azide control. At the indicated time points from 1–10 days, wells were aspirated and any adherent HLMC were removed using trypsin solution (Fisher Scientific, Loughborough, UK). Aspirated cells were transferred to a 96 well V bottom plate and centrifuged at 300 × g for 5 minutes. Supernatants were removed and stored at -20°C and cells were resuspended in 10 μl of DMEM. 10 μl of trypan blue solution was added to the cells and cell number and viability were assessed using a haemocytometer.

For the determination of pro-survival cytokine activity in the cultures, neutralising anti-human IL-6 (1 μg/ml) (mouse IgG_1_), anti-human SCF (1 μg/ml) (goat polyclonal IgG) or an isotype control (1 μg/ml) (mouse IgG_1_) antibodies (all from R&D, Abingdon, UK) were added to cultures containing 3 μg/ml IgE.

### Isolation of HLMC total RNA

2 × 10^6 ^HLMC were incubated for 24 hours in growth medium with or without 3 μg/ml IgE in 6 well plates. Cells were aspirated and centrifuged at 250 × g for 8 minutes. Supernatants were removed and the cells resuspended in 25 ml of sterile PBS before centrifuging again. Total RNA was then isolated using an SV Total RNA Isolation System (Promega, Southampton, UK) according to the manufacturer's instructions.

### Quantitative real-time RT-PCR

Quantitative real time RT-PCR was performed using a single tube Full Velocity SYBR Green Kit (Stratagene, Amsterdam, Netherlands). Primers were designed for IL-6 to span exon-exon junctions to eliminate DNA contamination issues. PCR products were designed to be between 200 and 300 base pairs in length in line with the optimum length for SYBR green use. The Full Velocity SYBR Green QRT-PCR kit uses a combined annealing/extension step of 60°C. Therefore, all primers were designed to have a T_m _within 1°C of 60°C. Primer3 software was used for the design of the primers [40]. Primer sequences for IL-6 were: forward primer 5'-GCACTGGCAGAAAACAACCT-3'; reverse primer 5'-CAGGGGTGGTTATTGCATCT-3'. The product length for IL-6 was 253 bp.

The QRT-PCR reaction was optimised for each gene and 200 nM final concentration of primers was assessed as the optimum for use over all conditions. Using this concentration of primers ensured no non-specific amplification occurred in the sample wells. Reverse transcription was carried out for 30 minutes at 50°C before 5 minutes at 95°C was used to inactivate the reverse transcriptase which otherwise interferes with the DNA polymerase. 50 cycles of 95°C for 20 seconds followed by 60°C for 30 seconds were carried out and fluorescence was collected at the end of each extension cycle using the Mx3000P QPCR machine (Stratagene). A SYBR green dissociation curve was carried out at the end of the reactions to ensure specificity and products were run on 1.5% agarose gels to determine product length. Products were gel purified (QIAGEN, Crawley, UK) and sequenced using the Protein and Nucleic Acid Chemistry Laboratory, University of Leicester.

For the comparative QRT-PCR, the internal normaliser gene used was β-actin and all mRNA expression data were normalised to β-actin and corrected using the reference dye (ROX). The β-actin primer sequences used were: forward primer – 5'-TTCAACTCCATCATGAAGTGTGACGTG – 3', reverse primer – 5'-CTAAGTCATAGTCCGCCTAGAAGCATT – 3'. The product length for β-actin was 310 bp. Only experiments where a distinct single peak was observed with a melting temperature different to that of the no template control were used. Expression data was expressed as the relative gene expression compared to the calibrator (unstimulated cells) as determined by the Mx3000P software's built in algorithm using an adaptive baseline to determine the C_t_.

### Measurement of IL-6 in HLMC supernatants

Supernatants removed from the survival assays were stored at -20°C prior to measurement of IL-6. Samples were measured for IL-6 from conditions following 7 days of cytokine withdrawal. IL-6 was measured using the Quantikine human IL-6 ELISA kit (R&D, Abingdon, UK) according to the manufacturer's instructions.

### Statistical analysis

Data is presented as the mean ± SEM unless otherwise stated. IL-6 release is presented as ng/10^6 ^cells. Differences between paired data were evaluated using Student's paired two-tailed *t *tests. Comparative quantitative RT-PCR data is presented as the relative expression compared to control (untreated cells) after correction against the internal calibrator β-actin as determined by the Mx3000P software's built in algorithm using an adaptive baseline to determine the C_t_. Due to the inherent variability of relative expression data according to the algorithm used, relative expression was also calculated using the 2^-ΔΔCt ^method [23] and the Relative Expression Software Tool (REST 2005, version 1.9.12, Corbett Life Science). In the untreated cells, a C_t _value was not always achieved so the final cycle (cycle 50) was assigned as the C_t _which tends to underestimate the degree of upregulation. Traditional statistical analyses for quantitative PCR are not appropriate due to high variability of gene expression in *ex vivo *human cells. Thus comparative QRT-PCR statistics were obtained by a hypothesis test with randomised bootstrapping of values with 50,000 iterations using the Relative Expression Software Tool (REST 2005, version 1.9.12, Corbett Life Science).

## Authors' contributions

GC designed and carried out the quantitative PCR, carried out survival assays, carried out IL-6 ELISA, performed statistical analyses and wrote the manuscript. SC carried out survival assays and blocking antibody experiments. PB conceived the study, and participated in its design and coordination and wrote and edited the manuscript. All authors read and approved the final manuscript.
